# Body Burden of Hg in Different Bio-Samples of Mothers in Shenyang City, China

**DOI:** 10.1371/journal.pone.0098121

**Published:** 2014-05-23

**Authors:** Min-ming Li, Mei-qin Wu, Jian Xu, Juan Du, Chong-huai Yan

**Affiliations:** 1 MOE-Shanghai Key Lab of Children's Environmental Health, Xinhua Hospital Affiliated to Shanghai Jiao Tong University School of Medicine, Shanghai, China; 2 Department of Gynecology and Obstetrics, Shengjing Hospital Affiliated to China Medical University, Shenyang, China; The Ohio State University, United States of America

## Abstract

Hg is an accumulative and neuro-toxic heavy metal which has a wide range of adverse effects in human health. However, few studies are available on body burden of Hg level in different bio-samples of pregnant women in Chinese population. Therefore, this study evaluated Hg levels in different maternal bio-samples in Shenyang city, China and investigated the correlation of Hg levels in different bio-samples. From October to December 2008, 200 pregnant women about to deliver their babies at ShengJing Hospital (Shenyang city, northeast of China) participated in this study. The geometric mean (GM) of Hg levels in cord blood, maternal venous blood, breast milk, and maternal urine were 2.18 µg/L, 1.17 µg/L, 1.14 µg/L, and 0.73 µg/L, respectively, and the GM of maternal hair Hg level was 404.45 µg/kg. There was a strong correlation between cord blood and maternal blood total Hg level (r = 0.713, P<0.001). Frequency of fish consumption more than or equal to 3 times per week during pregnancy was suggested as a significant risk factor of prenatal Hg exposure (unadjusted OR 3.5, adjusted OR 2.94, P<0.05). This study provides evidence about Hg burden of mothers and the risk factors of prenatal Hg exposure in Shenyang city, China.

## Introduction

The amount of global Hg emission is critical and environmental Hg contamination has emerged to become a public health concern, especially in Asia. In 1990, the Hg discharged in Europe accounted for 37% of the total amount, while China and Japan accounted for 28% and North America accounted for 13%, respectively. However, in 2000, Hg discharge in Asia quickly increased to 54%, while Africa accounted for 18% and Europe accounted for 11%, respectively [1]–[3]. China has become the largest coal producer and consumer in the world. Hg emission in China was up to 6 tons in 2003, with 44% due to coal combustion [4].

Hg is an omnipresent toxin which causes a wide range of adverse health problems in humans. As a neuro-toxic heavy metal, Hg destroys migration and maturation of fetal nerve cells, causes oxidative distress, damages cell membrane structure and impairs the synthesis of protein, such as neurotransmitters [5],[6]. Previous studies have revealed that Hg could be easily transmitted from animal mother to offspring through placenta by prenatal exposure of Hg vapor [7].

The prenatal period is believed to be the most susceptible stage of life and the fetus is considered to be most sensitive to Hg exposure [8]. Many studies have revealed that high prenatal exposure to Hg related to the poor performance of non-dominant hand grooved pegboard in male and high scores of hyperactivity index significantly [9],[10]. Even low-level prenatal exposure to Hg could impair the growth of fetus [11]–[13]. The follow-up study on the age of 7 in Faroe Islands cohort indicated that the prenatal MeHg (methyl Hg) exposure was strongly correlated with the decline of children's attention, linguistic capacity and memory [14]. Axelrad et al. found a dose-response relationship of prenatal Hg exposure and children's IQ (Intelligence Quotient) score: 1 ppm (1 mg/Kg) increase of maternal hair Hg level was correlated with 0.18 decreasing of the IQ Score [15].Thus, evaluation of Hg burden in pregnant women is critically important for offspring's growth. Hg levels in different bio-samples could reflect Hg exposure. A study in Japan revealed that geometric means of Total Hg (THg) in hair, cord tissue, venous blood and cord blood of 115 pregnant women were 1624, 91.7, 5.18, and 9.81 ng/g, respectively [16]. 8% of the breast milk samples provided from healthy mothers in Vienna, Linz, and Tulln exceeded the screening level of 3.5 µg/L for Hg [17]. A recent survey which included 1323 mother-neonate pairs from 7 administrative districts in China showed that the arithmetic mean and geometric mean of total Hg in umbilical cord blood were 2.26 µg/L and 1.81 µg/L, respectively, and the difference between those districts was statistically significant [18].

Nonetheless, few studies about Hg burden were obtained in different bio-samples of mothers to evaluate prenatal Hg exposure and examine the correlation between Hg levels of different bio-samples in Chinese population.

Thus, our study aimed to evaluate Hg levels of different bio-samples in Shenyang city (Liaoning province in northeast of China) and investigated the relationship of Hg levels in different bio-samples.

## Materials and Methods

This study is a cross-sectional study conducted in Shengjing hospital Shenyang city, Liaoning province, in northeast of China from October 2008 to December 2008. The study protocol was approved by the Medical Ethics Committee of Shanghai XinHua Hospital affiliated to Shanghai Jiao Tong University School of Medicine and the Medical Ethics Committee of Shengjing Hospital. We have obtained written informed consent from all study participants. All of the procedures were done in accordance with the Declaration of Helsinki and relevant policies in China.

### Subjects and samples

#### Sample size determination

According to the primary objective of this study, that was evaluation the body burden of Hg in different bio-samples of mothers, sample size was determined by the formula about continuous variable (N = [(Z_α_×σ)/δ]^2^). Standard deviations could be estimated through formula, which is range/6, from data of median, range and sample size, supplied by previous studies about maternal bio-sample Hg levels from China [19],[20].

#### Subject selection

This study was based on voluntary participation. 197 pairs of mother-newborns who signed the informed consent were included. Inclusion criteria include singleton pregnancy; 37–42 weeks of gestation and Apgar score no less than 8. Exclusion criteria included external fertilization case and unhealthy mothers or newborns with disorders such as liver and renal dysfunction, hypothyroidism, nervous and mental diseases, immunodeficiency, malignant tumors, hematological diseases, hypertension, cardiac diseases, diabetes mellitus, tuberculosis, severe preeclampsia, and gestational diabetes.

### Data collection

All participating mothers were required to complete a questionnaire about living environment. The information listed in the questionnaire about the demographic, socioeconomic characteristics and the personal living habits of the mothers was collected through an interview by specially trained investigators. Dietary information about eating frequency per week in the last three months before delivery was also collected to evaluate pregnant nutrition supplying and Hg intake from common food. Birth weight, length and head circumference were measured by trained nurses.

### Examination of Hg levels

Bio-samples were collected within 24 hours after delivery. Breast milk must be the colostrum collected in a few days after delivery. Mother's hair in the first 3 cm (close to scalp) was cut with stainless steel scissors. All samples were stored at −20°C immediately with serial number after collection.

The concentration of THg in bio-samples was detected by cold vapor atomic absorption spectrometry using an automatic Hg analyzer (Model DMA-80, Milestone, Italy). Hair samples were washed with distilled water firstly, followed by deionized water and then air-dried, and finally each 200 mg sample was placed into a nickel sample boat. Each 100 µL of blood sample was placed into one sample boat, then placed into the atomic absorption spectrometry instruments. Samples were analyzed using a standardized method [19].

The accuracy of detection was guaranteed by the use of standard reference material (Contox; Kaulson Laboratories, Inc., NJ, USA). An empty sample boat was detected regularly to ensure Hg was not cross-contaminated between samples. A new boat was used every 50 samples after twice heated to remove traces of Hg. The limit of detection (LOD) was 0.3 µg/kg for total Hg in maternal hair and 0.3 µg/L for total Hg in cord blood, maternal venous blood, breast milk, and maternal urine. No sample had an Hg level less than the LOD. The RSD% (Relative Standard Deviation) of repeating detection of controls and samples was controlled below 2%.

### Data analysis

IBM SPSS Statistics 20 (SPSS Inc., Chicago) was used for statistical calculation. Descriptive statistics were performed for each variable. The differences of birth weight, birth length and birth head circumference between two groups were determined by T-test of independent sampler. P-value less than 0.05 considered statistically significant. Spearman's correlation was used to evaluate the correlation of Hg levels between different bio-samples. Logistic regression analysis was used as multivariate analysis.

## Results

The study included 197 pairs of healthy mother-infant at Shengjing hospital in Shenyang city in northeast of China. All families enrolled in the study have signed the informed consent.

### Demographic characteristics

The mean age of these 197 mothers was 30.11 years old (range 21.77–41.87 years). Most of the mothers were Han people (88.00%) and lived in urban areas (87.00%). Most of the mothers (79.70%) had university or above degree. Household income per month (RMB, China currency) of more than half participated families was between 2000 to 5000 RMB (about 300–800$). Most of the mothers ate aquatic product <3 times per week, and rarely took dietary supplements such as calcium, iron and cod-liver oil during postconceptual age. Delivery mode of most mothers (89.53%) was section. For the newborns, the mean gestational age was 38.59 weeks (ranged from 32 to 42 months), the mean birth weight was 3422.16 g (ranged from 2171 g to 4950 g), the mean birth length was 50.70 cm (ranged from 45 cm to 56 cm), and mean head circumference was 34.28 cm (ranged from 30 cm to 39.5 cm). (Table 1)

**Table 1:** Characteristics of the Study Population.

Characteristic	Mean (SD) (range) or %	
Mothers		
Age, y	30.11(±3.97) (21.77–41.87)	
Pre-pregnancy BMI, kg/m^2^	21.7 (±3.68) (16.49–48.95)	
Race		
Han nationality	88%	
Minority nationalities	12%	
Residence		
Urban	87%	
Suburban	7%	
Rural	6%	
Education		
Up to primary	1.01%	
Secondary	19.29%	
University	79.70%	
Monthly household income per capita/month (RMB)		
<5000	49.75%	
5000–10000	37.56%	
10000–15000	6.6%	
>15000	6.09%	
Family members' occupation		
Hg related	26.60%	
Non-Hg	73.40%	
Using whitening cosmetic products		
Non or rarely	89.18%	
>3 times per week	10.82%	
Filling tooth during pregnancy	1.53%	
Mother smoking status		
Pre-pregnancy active smoking	5.64%	
Cigarette smoke exposure during pregnancy >3 time per week#	55.33%	
Dietary intake during pregnancy		
fresh fish intake^a^		
≤3 times per week (rough Hg intake Non or ≤22.5 µg per week)	90%	
>3 times per week (rough Hg intake >22.5 µg per week)	10%	
Shellfish intake^b^		
<1 time per week (rough Hg intake Non or <3.75 µg per week)	84.26%	
1–3 times per week (rough Hg intake 3.75–11.25 µg per week)	14.72%	
>3 times per week (rough Hg intake >11.25 µg per week)	1.02%	
Shrimp and crab intake^c^		
<1 times per week (rough Hg intake Non or <2.25 µg per week)	44.16%	
1–3 times per week (rough Hg intake 2.25–6.75 µg per week)	53.3%	
>3 times per week (rough Hg intake >6.75 µg per week)	2.54%	
Vegetable intake		
Non or ≤3 times per week	14.80%	
4–7 times per week	54.59%	
≥2 times per day	30.61%	
Fruit intake		
Non or ≤3 times per week	11.73%	
4–7 times per week	45.92%	
≥2 times per day	42.35%	
Dietary supplement intake during pregnancy	Non or rarely	Everyday
Calcium	75.63%	24.37%
Iron	87.23%	12.77%
cod-liver oil	96.34%	3.66%
Type of born		
Normal spontaneous delivery	10.47%	
Section	89.53%	
Newborns		
Gestational age (m)	38.59±1.08 (32–42)	
Gender		
Female	45.41%	
Male	54.59%	
Birth weight (g)	3422.16±433.38(2171–4950)	
Birth length (cm)	50.70±2.14 (45–56)	
Birth head circumference (cm)	34.28±1.92 (30–39.5)	

a rough Hg intake from fish was evaluated by formula: adult RNI of fish (about 75 g/day) × Hg content of fish in this area (about 0.1 mg/kg).

b rough Hg intake from shellfish was evaluated by formula: adult RNI of aquatic product (about 75 g/day) × Hg content of shellfish in this area (about 0.05 mg/kg).

c rough Hg intake from shrimp and crab was evaluated by formula: adult RNI of aquatic product (about 75 g/day) × Hg content of shellfish in this area (about 0.03 mg/kg).

# cigarette smoke exposure concluded active smoking and passive smoking.

### Hg concentration in different bio-samples

The geometric mean, 95% confidence interval and 10, 25, 50, 75, and 90 percentiles of Hg concentration in umbilical cord serum (n = 195), maternal venous blood (n = 192), breast milk (n = 195), maternal hair (n = 179) and maternal urine (n = 195) were shown in table 2.

**Table 2:** Concentration of Hg in different bio-samples.

Bio-samples	n	GM (95% CI)	maximum	minimum	10th P	25th P	50th P	75th P	90th P
umbilical cord blood (µg/L)	195	2.18 (2.06, 2.31)	5.09	0.77	1.24	1.64	2.15	2.95	3.83
maternal venous blood (µg/L)	192	1.17 (1.10, 1.24)	3.83	0.26	0.82	0.85	1.24	1.42	2.04
breast milk (µg/L)	195	1.14 (1.03, 1.26)	8.40	0.42	0.43	0.85	0.97	1.72	3.19
maternal hair (µg/Kg)	179	404.45 (376.79, 434.14)	1905.97	92.6	229.51	296.47	393.90	554.76	796.66
maternal urine (µg/L)	195	0.73 (0.68, 0.79)	5.44	0.06	0.42	0.45	0.82	0.85	1.26

P: percentile.

### The socioeconomic characteristic and living habit of mothers in different groups

Mothers with umbilical cord Hg level less than or equal to 75% were in group 1 (n = 150); Hg level above 75% were in group 2 (n = 45). The family socioeconomic information, mother's living habits, food and supplement intake in the last three months before delivery were shown in table 3. Hg intake of aquatic product was estimated according to aquatic product intake of mothers and Hg content of aquatic product in this area. Mother's aquatic product intake is about 75 g per day, estimated by recommended nutritional intake (RNI) of aquatic product according to dietary guideline of Chinese inhabitants [21]. Previous study supplied information about Hg contents of food in China. Hg contents of fresh fish, shrimp and crab, shellfish in this area were 0.1 mg/Kg, 0.03 mg/Kg, 0.05 mg/Kg respectively [22].

**Table 3:** Characteristics of mothers with cord blood Hg level less than or equal to 75% (n = 150, Group 1) and above 75% (n = 45, Group 2).

Characteristic	Group 1: umbilical cord blood Hg level ≤75 Percent (N = 150)	Group 2: umbilical cord blood Hg level >75 Percent (N = 45)
Residence		
Urban	128(85.3%)	43(95.6%)
Non-urban	22(14.7%)	2(4.4%)
Mother's education		
Under or reach senior middle school	36(24%)	3(6.7%)
Above senior middle school	114(76%)	42(93.3%)
Family month income (RMB)		
<5000	80(53.3%)	18(40%)
≥5000	70(46.7%)	27(60%)
Family member's occupation		
Hg related	44(29.3%)	8(17.8%)
Non-Hg	106(70.7%)	37(82.2%)
Pre-pregnancy Active smoking		
No	143(95.3%)	41(91.1%)
Yes	7(4.7%)	4(8.9%)
Cigarette smoke exposure during pregnancy >3 time per week		
No	70(46.7%)	16(35.6%)
Yes	80(53.3%)	29(64.4%)
Using whitening cosmetic products		
Non or ≤3 times per week	135(90%)	40(88.9%)
>3 times per week	15(10%)	5(11.1%)
Dietary intake during pregnancy		
Fresh fish intake		
≤3 times per week (rough Hg intake Non or ≤22.5 µg per week)	140(93.3%)	36(80%)
>3times per week (rough Hg intake >22.5 µg per week)	10(6.7%)	9(20%)
Shellfish intake		
<1 time per week (rough Hg intake Non or <3.75 µg per week)	129(86%)	37(82.2%)
≥1 times per week (rough Hg intake ≥3.75 µg per week)	21(14%)	8(17.8%)
Shrimp and crab intake		
<1 time per week (rough Hg intake Non or <2.25 µg per week)	87(58%)	18(40%)
≥1 times per week (rough Hg intake ≥2.25 µg per week)	63(42%)	27(60%)
Vegetable intake		
<2 times per day	108(72%)	28(62.2%)
≥2 times per day	42(28%)	17(37.8%)
Fruit intake		
<2 times per day	89(59.3%)	24(53.3%)
≥2 times per day	61(40.7%)	21(46.7%)
Birth weight (g)	3350.33±684.89	3345.51±674.08
	P = 0.93	
Birth length (cm)	49.341±8.4	47.53±13.01
	P = 0.04	
Birth head circumference (cm)	34.47±1.67	34.62±1.71
	P = 0.99	

In group 1, the averages of birth weight, birth length and head circumference of the newborns were 3350.33 g, 49.34 cm and 34.47 cm, respectively, while in group 2, they were 3345.51 g, 47.53 cm and 34.62 cm, respectively. Birth length between the two groups was different significantly (p = 0.04).

### Correlation of Hg levels between different bio-samples

**Figure 1:**
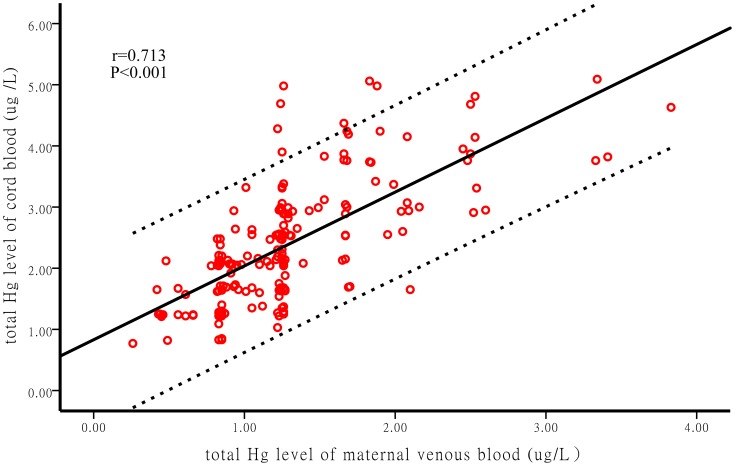
Correlation of Hg levels between umbilical cord blood and maternal venous blood, dotted lines represent 95% confidence interval of Spearman correlation coefficient.

Correlation coefficient among the Hg concentrations in umbilical cord blood, maternal venous blood, hair, breast and urine in Shenyang province of China were shown in table 4. THg level in cord blood showed strong correlation with those in maternal blood as shown in Table 4. (r = 0.713, P<0.001, Fig. 1).

**Table 4:** Spearman correlation coefficient of overall Hg level in umbilical cord blood, maternal venous blood, hair, breast and urine in Shenyang province, China.

	umbilical cord blood	maternal venous blood	Maternal urine	Breast	Maternal hair	Urine Hg/Creatinine
umbilical cord blood	1.000	0.713*	0.234*	0.173*	0.278*	0.059
maternal venous blood		1.000	0.208*	0.088	0.180*	0.041
Maternal urine			1.000	−0.050	0.014	0.420*
breast				1.000	−0.042	−0.056
Maternal hair					1.000	0.058
Urine Hg/Creatinine						1.000

*means p-value<0.05

### Risking factors of cord blood Hg level above median

Mother's family socioeconomic characteristic, living and eating habits were analyzed to evaluate their effect on cord blood Hg level. The impact of fresh fish consumption frequency >3 time per week (rough Hg intake >22.5 µg per week) during postconceptual age was significant (unadjusted odds ratio (OR) 3.5, adjusted OR 2.94, P<0.05). The impact of shrimp and crab consumption frequency ≥1 time per week (rough Hg intake ≥2.25 µg per week) was significant with unadjusted OR 2.07 (P = 0.04), but not significant when adjusted by residence area, mother's education level, family month income, family member's occupation related with Hg or not, using frequency of whitening cosmetic products, frequency of green vegetable and fruit intake during pregnancy (adjusted OR 1.78, P = 0.11). The result did not show significant impact of pre-pregnancy smoking and cigarette smoke exposure (active smoking and passive smoking) during pregnancy, shellfish consumption on increasing cord blood Hg level. (Table 5)

**Table 5:** Logistic regression analysis: factors affecting the umbilical cord blood Hg level.

Variables	Unadjusted			Adjusted^a^			Power
	Odds ratio	95% CI of OR	P value	Odds ratio	95% CI of OR	P value	
Pre-pregnancy active smoking	1.99	0.56–7.14	0.29	3.07	0.68–13.83	0.14	31%
Cigarette smoke exposure during pregnancy >3 time per week	1.59	0.8–3.16	0.19	1.68	0.82–3.46	0.16	38%
Frequency of fresh fish consumption during pregnancy >3 time per week (Hg intake ≥22.5 µg per week)	3.5	1.32–9.25	0.01	2.94	1.08–8.03	0.04	60%
Frequency of Shrimp or crab consumption during pregnancy ≥1 time per week (Hg intake ≥2.25 µg per week)	2.07	1.05–4.08	0.04	1.78	0.88–3.61	0.11	62%
Frequency of Shellfish consumption during pregnancy ≥1 time per week (Hg intake ≥3.75 µg per week)	1.33	0.54–3.24	0.53	1.28	0.50–3.29	0.61	8%

a adjusted by residence area, mother's education level, family month income, family member's occupation related with Hg or not, using frequency of whitening cosmetic products, frequency of green vegetable and fruit intake during pregnancy.

## Discussion

Prenatal Hg exposure could be evaluated by Hg concentration in bio-samples from pregnant women. Hg levels in venous blood and umbilical cord blood was suggested to serve as a good indicator of prenatal Hg exposure in post-conceptual age [23],[24]. Similarly, Hg levels in hair, urine, breast milk, or cord tissue of mother and newborn's meconium also function as good parameters to reflect Hg exposure during pregnancy [25]. Urine Hg level reflects cumulative Hg exposure over recent months, because the half-life of urine Hg is 40-60 days [26]–[29]. Furthermore, the Hg level of cord blood and venous blood were correlated with hair Hg level which could reflect Hg exposure. The prenatal Hg exposure was also suggested to lead to increased Hg level in breast milk since Hg could be excreted through breast milk [17].

In this study, GM of Hg concentration in umbilical cord blood, maternal venous blood, breast milk, hair and urine of mothers in Shenyang city were 2.18 µg/L, 1.17 µg/L, 1.14 µg/L, 404.45 µg/Kg, and 0.73 µg/L, respectively. A Survey in Japan had revealed that THg levels in hair, maternal venous blood and cord blood of 115 pregnant women were 1624, 5.18, and 9.81 ng/g, respectively, much higher than these in our study and also much higher than geometric mean level of Hg in women of child-bearing age (16–49 years old) in US according the 1999–2000 data from the National Health and Nutrition Examination Survey in US [16],[30]. This result was believed to be largely attributed to dietary habits in Japan. Many studies have shown that more frequently fish or shellfish consumed related to higher Hg levels in bio-samples [19],[31]–[33]. Japanese people consume much more fish and shellfish than the population examined in our study from Shenyang city, an inland city in northeast of China. The Hg level in our study was also lower than the level of the population in Zhoushan city (the GM of Hg level in cord blood and maternal hair were 5.58 µg/L, and 1246.56 µg/kg, respectively), which might be attributed by high risk of Hg exposure in population from coastal city in southeastern China [19]. Larger consumption of marine product in population of Southeastern China as well as higher Hg content of marine product from southeastern China than those of north China might lead to the more opportunity of Hg exposure [19],[22].

As for the relation of prenatal Hg exposure and birth outcome, in the cohort of pregnant women in Spain, high cord blood concentration of THg was associated with reduced birth weight and birth length, and an increased risk of being born SGA (small for gestational age) for length, but not weight [34]. In this study, result showed significant difference in terms of birth length but no statistical difference in terms of birth weight and head circumference between the groups with umbilical cord blood Hg level ≤75% or >75%. The birth length in group with high cord blood Hg level was shorter compared to the group with relative low cord blood Hg level. Geometric mean concentration of cord blood Hg in this study was 2.18 µg/L much lower than that in Spain cohort (9.4 µg/L) and also lower than the limit accepted by US Environmental Protection Agency (EPA), which might explain the lack of statistic difference on birth weight and head circumference [34],[35]. Also, the amount of fish intake during pregnancy which was proved by several studies as a risk factor of Hg exposure and adverse birth outcome, such as SGA, was less than that in Spain cohort population [34],[36]–[39].

Furthermore, the Hg level of the cord blood in this study was higher than level of the maternal blood, which was in agreement with the findings in Spain, Poland, Greenland, Korea and Japan [16],[39]–[41]. It can be explained by the accumulation of Hg in the body. Hg exists in different forms including metallic Hg, inorganic Hg and organic Hg. The metallic Hg remains bound to hemoglobin and glutathione in the red blood cells in the body. Naturally, fetuses have higher hemoglobin level than adults and are more liable to Hg accumulation [16],[42].

As for impact of mother living and dietary habits on increasing umbilical cord blood Hg level, a series of variables such as family socioeconomic status, mother's living habit, frequency of vegetable, fruit and dietary supplement intake, aquatic product intake during postconceptual age were taken into account accordingly.

In this study, the ration of pre-pregnancy smoking and cigarette smoke exposure above 3 times per week during pregnancy in group with high cord blood Hg level was higher than in group with low cord blood Hg level. However, the impact of pre-pregnancy smoking and smoke exposure during pregnancy on prenatal Hg exposure were not found significantly, even adjusted by residence area, mother's education level, family month income, family member's occupation related with Hg or not, frequency of using whitening cosmetic products, frequency of green vegetable and fruit intake during pregnancy (p>0.05). In fact, many heavy metals such as cadmium, chromium, copper, lead, Hg, nickel, and zinc are all found in tobacco, cigarette paper, filters, and cigarette smoke [43]. A study found that Hg contents in tobacco from different brands ranged from 2.95 to 10.2 ng Hg per cigarette and almost all Hg contained in cigarette was released to the smoke (from 86.7 to 100%) [44]. Smoke is inhaled into lung via respiratory tract, diffuses among pulmonary alveoli, and then enters blood through alveolar - capillary membrane. Hg in blood is bound to hemoglobin and glutathione in the red blood cells. Some Hg penetrates through brain-cortex and placenta barrier, which causes Hg deposition in embryo brain and other tissues [45]. As for impact of cigarette smoke on prenatal Hg exposure, the results of many studies were not consistent. A previous study showed smoking during the pregnancy and increasing number of cigarettes elevated mother Hg levels during pregnancy at statistically significant rate (p<0.05) [46]. Another study revealed that blood Hg concentration tended to be higher in smokers, but the trend was not statistically significant [47]. Many studies have not found a significant correlation between the mother smoke exposure status and cord blood Hg levels [48],[49]. In this study, the test power in analyzing impact of smoke exposure through multiple logistical regression was small, which might result from small sample size limitation.

In this study fresh fish intake more than 3 times per week (Hg content more than 22.5 µg/L) during postconceptual age had significant effect on cord blood Hg level. This result was similar to many previous studies, which showed that increased intake of canned tuna, lean fish, and large oily fish by pregnant women was associated with elevated THg concentration in cord blood and intrauterine growth retardation [34],[36]–[38]. Many study revealed that aquatic animal could accumulate OHg (organic Hg) in water and deliver MeHg to human through food chain [50]–[52]. Consumption of fresh fish is a major route of human exposure to Hg [53]–[55]. Concentration of OHg in fish could reach 10^4^–10^6^ times of Hg level in water through biological magnification [50]. OHg is apt to be absorbed by body, to penetrate through brain-cortex and placenta barrier and cause neurotoxicity [45]. A US cohort in 1999–2002 revealed that maternal fish intake was correlated with erythrocyte total Hg (Spearman's r = 0.42, p<0.0001), with an unadjusted increase of Hg level 0.94 ng/g for each weekly fish serving [56]. A research showed consumption of canned and fresh market fish was significantly related to cord blood OHg levels. While blood IHg (inorganic Hg) levels were not found related to fish consumption prior to and during pregnancy [57]. Thus, some subsequent advisories and expert reviews have still continued to recommend that pregnant women consume no more than two fish servings per week, even considering the nutrients supplied by fish, such as (n-3) PUFA (polyunsaturated fatty acid) which was approved to be benefit to infant brain development[58],]59]. However, other study revealed higher fish intake (fish intake of >2 servings/week) was associated with better child cognitive test performance [56]. Thus, recommendations for aquatic product intake during pregnancy should take into account the nutritional benefits as well as the heavy metal potential harms.

This study showed that THg level in cord blood was significantly related to the level in maternal venous blood. This result was similar to previous studies performed in Korean, Japanese and Philippines population [16],[60],[61]. Sakamoto found that individual cord/maternal Hg concentration in red blood cells varied from 1.08 to 2.19 in 53 mother–infant pairs [62]. Stern and Smith summarized the variability of cord/maternal blood Hg level ratio and suggested that the cord: maternal ratio calculated from MeHg-only was larger than the ratio calculated from THg [63]. The fact of increased concentration of MeHg in cord blood relative to maternal blood was proved to result from larger hematocrit and greater hemoglobin concentration in the newborn, considering the greater MeHg binding capacity in fetal hemoglobin [64].

In the present study, the correlation of THg in maternal hair and other biomarkers was comparatively low, that was similar to a previous study [16].

Previous study showed a relationship between level of total Hg in maternal blood and in breast milk (r = .66, p = .0001), with the average breast milk level as 27% of the blood levels [65]. However, weak correlation was detected between the Hg level of breast milk and other bio-samples in this study. This might result from the fact that amount of variables including area of residence, prematurity, consumption of cereals and vitamin supplementation were suggested to affect Hg level in breast milk [66]. Pesch found that there was a strong correlation between urinary Hg level and 24 h total volume of urine (r = 0.866). An even stronger correlation was found between the urinary Hg level and creatinine excretion (r = 0.933) [67]. In this study, urinary Hg concentration related to creatinine was applied as standardized urinary Hg level. As shown in this study, the correlation of the Hg level in urine and Hg level in other bio-samples were rather low, which was similar to the result of previous studies [67]. This might be explained by the complicated mechanism of Hg absorption and excretion in kidney [68]. Too many influence factors affecting urinary Hg level, such as activity of γ-GT (gamma glutamyltransferase) at cell membrane and organic anion transports system at basement membrane of epithelial cells at proximal renal tubular, might also contribute to low relationship between urinary Hg level and Hg level of other bio-samples [69]. Olstad found a significant positive correlation (r = 0.55) of urine Hg and extent of amalgam restorations [70]. Two predictors were found to contribute to the variance of Hg levels in urine: the number of teeth with amalgam fillings account for 23.2% and the number of defective amalgam fillings accounting for 2.1% [67].

In this study, detailed information about living habit during pregnancy was collected by one-to-one interview, which could ensure accuracy of data. We also collected multiple types of bio-samples from mothers to better evaluate prenatal Hg burden. Hg exposure level from food were estimated by analyzing data of Chinese Dietary Reference Intakes (DRIs), Hg content of food in this area and food frequency, which could estimated Hg intake from different types of food.

Our study also has some limitations. Firstly, this study was not a randomized study, which might influence wide appliance of the conclusion. Secondly, for multiple logistical regression analysis, the sample size of this study was not large enough, which might lead to low power of test in analyzing risk factors of increasing cord blood Hg level. Thirdly, information about specific types of fish intake were not included in this study, thus the effect of different types of fish on the Hg exposure became unable to be analyzed. The fourth, frequency of eating per week instead of serving intake of each food was applied in this study to evaluate nutrient intake. Considering individual difference, precise intakes of different food were failed to be assessed only from eating frequency per week. Three days dietary retrospect may be the better method for measuring precise food intake. Lastly, this study explored only THg level of bio-samples in mother without analyzing different types of Hg such as MeHg, which accounts for over 80% of hair THg level in fish-eating populations [71],[72].

In summary, this study showed that Hg burden of pregnant women in Shenyang city was largely under the lower limit accepted by US EPA. There was a strong correlation between cord blood and maternal blood THg levels. Increased prenatal Hg exposure was associated with mothers' fresh fish consumption during pregnancy.
